# Where are the fastest master butterfly swimmers competing in the FINA World Masters Championships from?

**DOI:** 10.17179/excli2023-6199

**Published:** 2023-07-08

**Authors:** Katja Weiss, Aldo Seffrin, Marilia Santos Andrade, Wais Ahmad, Pedro Forte, Sascha Moreitz, Arkadiusz Stanula, Ivan Cuk, Pantelis T. Nikolaidis, Beat Knechtle

**Affiliations:** 1 Institute of Primary Care, University of Zurich, Zurich, Switzerland; 2 Postgraduate Program in Translation Medicine, Federal University of São Paulo, São Paulo, Brazil; 3 Department of Physiology, Federal University of São Paulo, São Paulo, Brazil; 4 Sanatorium Kilchberg, Kilchberg, Switzerland; 5 CI-ISCE, Higher Institute of Educational Sciences of the Douro, Penafiel, Portugal; 6 Department of Sports Sciences, Instituto Politécnico de Bragança, Bragança, Portugal; 7 Research Center in Sports, Health and Human Development, Covilhã, Portugal; 8 Radiology and Neuroradiology, Institute of Radiology, Spital Zollikerberg, Zurich, Switzerland; 9 Institute of Sport Sciences, Department of Swimming and Water Rescue, Academy of Physical Education in Katowice, Katowice, Poland; 10 Faculty of Sport and Physical Education, University of Belgrade, Belgrade, Serbia; 11 School of Health and Caring Sciences, University of West Attica, Athens, Greece; 12 Medbase St. Gallen Am Vadianplatz, St. Gallen, Switzerland

**Keywords:** swimming, performance, nationality, origin, records

## Abstract

While the butterfly stroke has received considerable attention in sports science, the origin of the fastest master butterfly swimmers remains unknown. The present study investigated which geographical locations produce the top-performing master butterfly swimmers within their age groups and gender. A total of 26,512 master butterfly swimmers (11,288 women and 15,224 men) competed in 50 m, 100 m and 200 m races in World Masters Championships held between 1986 and 2019. From each swimmer, the year of competition, first name, last name, age group and distance were recorded. Descriptive data were presented using mean, standard deviation, maximum and minimum values, and/or confidence intervals. The top 10 race times for master butterfly swimming and gender were identified for descriptive purposes. Nationalities were then grouped into six categories: the top five nationalities with the most appearances in the top 10 fastest times in butterfly swimming by distance each year and one group consisting of all other nationalities. In the event of a tie, the nationality with the most participants overall was selected. Generalized linear models (GLMs) with a gamma probability distribution and log link function were used to assess the effect of age groups and gender on swimming time. In summary, Germany had the fastest women butterfly master swimmers across all distances, while the USA had the fastest men butterfly master swimmers for all distances. Men covered all distances faster than women and younger swimmers were quicker than older swimmers. The results of this study can be utilized to determine the countries that produce the most successful master butterfly swimmers, providing a foundation for further research to explore the factors that lead to their success.

## Introduction

Swimming has been considered a competitive sport for centuries. The first known swimming races were held in Japan in the first century BCE (before the Common Era), and swimming was also a popular sport in ancient Greece and Rome (IOC, https://olympics.com/). The early Olympic Games included swimming events in open water rather than pools (olympics.com). 

Modern competitive swimming as we know it today developed in the 19^th^ century (olympics.com). The National Swimming Society was founded in England in 1837 and was one of the first swimming organizations in the world (https://www.eastswimming.org/). Its main purpose was to promote swimming as a sport and to organize swimming competitions. The society was responsible for organizing the first swimming championship in Australia in 1846, and it played a crucial role in developing swimming as a competitive sport worldwide (Encyclopedia Britannica[25]).

While the National Swimming Society is a historical organization with a focus on promoting swimming as a sport and organizing competitions at the national level, the International Swimming Federation (Fédération Internationale de Natation/FINA) is a global organization responsible for overseeing and regulating aquatic sports, including swimming, diving, water polo, synchronized swimming and open water swimming at an international level (https://www.worldaquatics.com/). It was founded in 1908 and currently has 209 member federations from around the world (worldaquatics.com). In January 2023, the federation underwent an official name change and became known as World Aquatics (worldaquatics.com).

The world of swimming boasts some of its biggest events through FINA, including the prestigious FINA World Championships and the FINA World Cup (worldaquatics.com/competitions). These competitions attract the world's best swimmers to showcase their skills and compete in various events and disciplines. 

Masters swimming, on the other hand, is a unique category of competitive swimming designed for individuals 25 years and older (worldaquatics.com/masters). The inaugural FINA World Masters' Championships took place in 1986, with the competitions featuring 14 age categories for swimmers ranging from 25 to 100 years old (worldaquatics.com/ masters). Notably, FINA stands as the only International Federation that recognizes world records for masters events in swimming (worldaquatics.com/masters). 

Swimming has undergone significant changes over the years (Mountjoy et al., 2016[57]). The competition has become more intense, and athletes are continuously pushing themselves to achieve new records and reach new limits (Nevill et al., 2007[58]; Pelayo and Alberty, 2011[64]). Research in competitive swimming has concentrated mostly on physiological, bioenergetics, biomechanics (Costa et al., 2010[16]; Feitosa et al., 2022[27]) aspects and lesson performance analysis (Costa et al., 2012[15]) and training characteristics (Amara et al., 2022[4]; Gourgoulis et al., 2019[32]). In individual sports, case studies present detailed information about a specific top swimmer field (Garland Fritzdorf et al., 2009[31]), identifying determinant factors. For that reason, it is important to identify the origin of top-performing swimmers to understand the factors that explain their performance. 

The increase in performance levels has been attributed to advancements in technology and sports science and changes in the approach to training and competition. Adopting more specialized training programs and using data analysis and tracking tools have enabled swimmers to train more effectively and efficiently (Berthelot et al., 2015[9]; O'Connor and Vozenilek, 2011[62]).

There are four swimming techniques: freestyle, breaststroke, backstroke and butterfly (Moser et al., 2020[56]). The butterfly started in the 1930s when it developed as a style of swimming breaststroke (Maglischo, 2003[48]). This stroke technique caught swimmers' and coaches' attention because they realized that breaststroke was quicker when a swimmer recovered their arms forward above the water and the arm technique - as well as the swimming term 'butterfly' - was born. It was established as an individual stroke by FINA in 1952 (worldaquatics.com). The butterfly stroke is a symmetrical swimming technique involving equal movements from both sides of the body, making it more demanding on the swimmer and less efficient in energy expenditure (Barbosa et al., 2006[5]). It requires both technical skill and physical endurance (Barbosa et al., 2008[6]; de Jesus et al., 2012[19]). Compared to front crawl and backstroke, butterfly presents larger fluctuations of velocity during the stroke cycle (Craig and Pendergast, 1979[17]). In addition, the energy cost of butterfly was lower than breaststroke and higher than front crawl and backstroke in elite swimmers (Capelli et al., 1998[12]). Due to the high energy requirements and low economy of movement, the butterfly stroke is typically limited to distances of 200 meters or less (FINA, 2017[29]). Scientific research on butterfly swimming focused mainly on technical details such as race kinematics (Morais et al., 2022[54]), stroke determinants (Barbosa et al., 2005[7]), arm-leg coordination (Seifert et al., 2008[70]), anthropometric characteristics (Alves et al., 2022[3]), velocity (Morais et al., 2021[53]) and pacing (McGibbon et al., 2018[51]).

The butterfly was contested at the Olympic Games for the first time in 1956, with a men's 200 m Butterfly event and a women's 100 m Butterfly event in Melbourne (IOC, olympics.com/swimming; worldaquatics.com). While only the 100 m and 200 m Butterfly events are contested at the Olympic Games (olympics.com/swimming), a 50 m Butterfly event is held at World and continental levels (worldaquatics.com/competitions). 

There is limited information available regarding the role of nationality among master swimmers (Born et al., 2022[11]). Although nationality has been well studied in the open-water swimming field (Knechtle et al., 2014[44]; Nikolaidis et al., 2018[59][60]; Seffrin et al., 2021[69]), little information exists on swimming distances. In the same direction, despite the aging effect on swimming performance in the open-water race has been already studied, there is no data about the aging effect in butterfly swimming. As butterfly presents a higher energy demand than freestyle, it is possible that the aging effect could be significantly intensified. The geographical origins of the top-performing master butterfly swimmers in their respective age groups and genders remain unknown. Therefore, the present study aims to investigate and identify the specific geographical locations associated with producing the top-performing master butterfly swimmers. We hypothesized that the United States would produce the fastest master swimmers, men would have better times than women in the same age group, and younger categories would have better times overall.

## Methods

### Ethical approval

This study was approved by the Institutional Review Board of Kanton St. Gallen, Switzerland, with a waiver of the requirement for informed consent of the participants as the study involved the analysis of publicly available data (EKSG 01/06/2010). The study was conducted following recognized ethical standards according to the Declaration of Helsinki adopted in 1964 and revised in 2013.

### Data set and data preparation

The race data were obtained from the official website of World Aquatics, formerly FINA (www.worldaquatics.com). We obtained full data from all World Masters Championships held between 1986 and 2019 (www.worldaquatics.com/masters/archives/masters-archives). A total of 26,512 swimmers (11,288 women and 15,224 men) competed in 50 m, 100 m, 200 m races between 1986 and 2019. From each swimmer, the year of competition, first name, last name age, age group and distance were recorded. Age groups were formed by grouping individuals every 5 years starting from the age of 25, ranging from 25-29, 30-34, 35-39, 40-44, 45-49, 50-54, 55-59, 60-64, 65-69, 70-74, 75-79, to 80 years or older (80+). Nationalities were then grouped into six categories: the top five nationalities with the most appearances in the top 10 fastest times in butterfly swimming by distance each year and one group consisting of all other nationalities.

### Statistical analysis

Descriptive data were presented using mean, standard deviation, maximum and minimum values, and/or confidence intervals. The top 10 race times for each swimming distance and gender were identified for descriptive purposes. The data did not follow a normal distribution or exhibit homogeneous variances, as determined by Shapiro-Wilk and Levene's tests, respectively. Therefore, the Kruskal-Wallis H test was used to compare differences between nationalities, and multiple pairwise comparisons adjusted by Bonferroni correction were performed to identify differences. Generalized linear models (GLMs) with a gamma probability distribution and log link function were used to assess the effect of age groups and gender on swimming time. Differences found were investigated with the posthoc Bonferroni test, and possible interactions with age group and gender were also explored. The Akaike Information Criterion (AIC) was used to choose the distribution of the dependent variable and the model linking function based on its lowest value (Akaike, 1974)[1]. The Omnibus test was also used to ensure that the model outperforms the null model. The significance level was set at 0.05, and SPSS version 26.0 (SPSS, Inc., Chicago, IL, USA) was used for all statistical analyses.

## Results

In master butterfly swimming, most swimmers (53.1 %) competed in the 50 m event (n=14,090, 5,810 women and 8,280 men), followed by the 100 m event (28.2 %, n=7,484, 3,205 women and 4,279 men), and the 200 m event (18.6 %, n=4,938, 2,273 women and 2,665 men). A ratio between the number of women and men competitors in each swimming distance and age group was calculated for descriptive purposes, which is presented in Table 1(Tab. 1).

Table 2(Tab. 2) displays the meantime, standard deviation, minimum, and maximum of each swimming distance for each gender.

**Figure 1:**
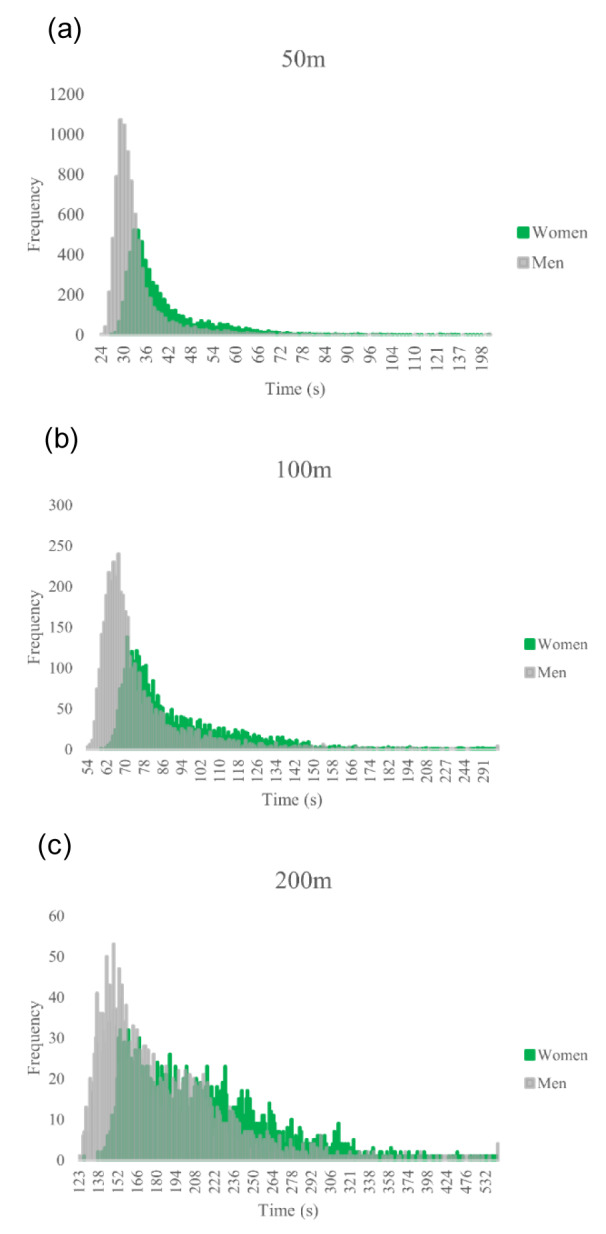
Race times histograms for women and men in all swimming distances ((a) 50 m, (b) 100 m, (c) 200 m). Total frequency in each swimming distance (a) n=14,090 (5,810 women and 8,280 men), (b) n=7,484 (3,205 women and 4,279 men), and (c) n=4,938 (2,273 women and 2,665 men)

Figure 1(Fig. 1) displays the histograms of race times for women and men in all swimming distances.

**Figure 2:**
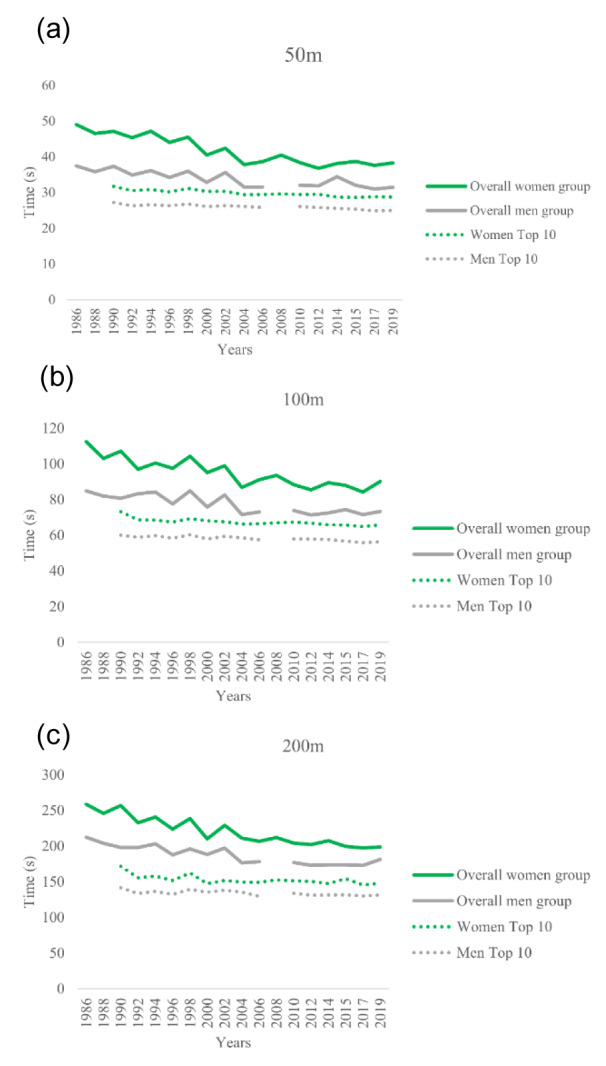
The mean time for each swimming distance ((a) 50 m, (b) 100 m, (c) 200 m)) for both women and men overall group and the mean times achieved by the top 10 athletes annually (Top 10) by gender. There was no men's data available for 2008 due to the unavailability of information in the database.

Figure 2(Fig. 2) presents the mean time for each swimming distance for both men and women overall group and the mean times achieved by the top 10 athletes annually. It is important to note that the top 10 fastest times for the year 1988 were excluded in Figure 2(Fig. 2) as they were not part of the nationality analysis due to the unavailability of information in the database. Additionally, there was no men's data available for 2008.

To compare the performance of the countries that participated in each swimming distance event, the five nationalities with the highest number of participants in the top 10 fastest times by year for each gender were selected. The other countries were grouped into a single category called “Others”. The mean time of all top 10 swimmers for each year, gender and country were compared and are displayed in Tables 3(Tab. 3) (women) and 4(Tab. 4) (men).

The country with the highest number of women swimmers (Table 3(Tab. 3)) in the top 10 fastest times by year was Germany for all butterfly distances. Meanwhile, the country with the highest number of men swimmers (Table 4(Tab. 4)) among the top 10 fastest times per year was the USA in all butterfly swimming distances. In addition to Germany, the USA and Great Britain were among the countries that most frequently reached the top 10 by year in all women's butterfly distances (50 m, 100 m, and 200 m) (Table 3(Tab. 3)). Conversely, the USA, Germany, and Great Britain were the countries that were among the top 10 in men's butterfly for all distances (Table 4(Tab. 4)). There were no differences between the countries in performance in the 100 m and 200 m butterfly for both women and men samples. However, for the 50 m butterfly, Russia presented a better performance than Great Britain for women (Table 3(Tab. 3)) and a better performance than the USA for men (Table 4(Tab. 4)).

**Figure 3:**
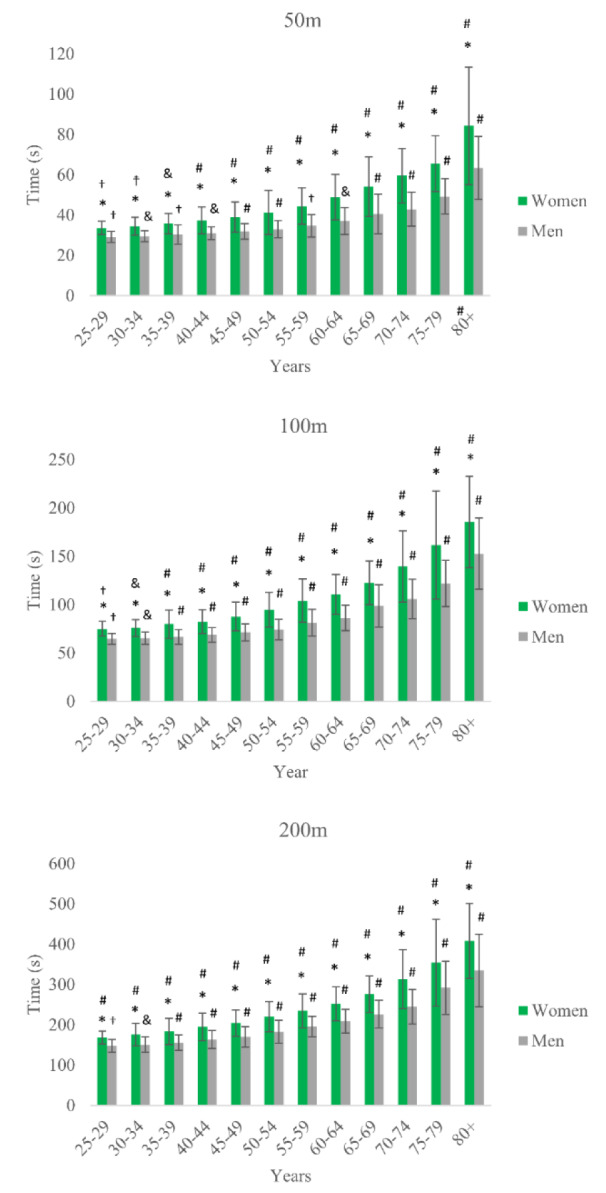
Race times by age group and gender * Different from the corresponding age group of the opposite gender; # Different from all other age groups of the same gender; & Different from all age groups of the same gender, except the immediately younger one; † Different from all age groups of the same gender, except the immediately older one; ☨ Different from all age groups of the same gender, except the immediately older and younger ones.

The results from the generalized linear models indicate that gender, age group, and the interaction between gender and age group all had significant effects on the 50 m, 100 m, and 200 m races. Specifically, for the 50 m races, the effect of gender was significant (x^2^ (1) = 3390.8, p < 0.001), as was the effect of age group (x^2^ (11) = 12835.9, p < 0.001), and the interaction between gender and age group (x^2^ (11) = 318.5, p < 0.001). The quality of the model was assessed using the Omnibus test (p < 0.001) and the Akaike Information Criterion (AIC = 89772.2). For the 100 m races, similar results were observed with a significant effect of gender (x^2^ (1) = 2338.0, p < 0.001), age group (x^2^ (11) = 13007.7, p < 0.001), and the interaction between gender and age group (x^2^ (11) = 157.8, p < 0.001). The model was superior to the null model according to the Omnibus test (p < 0.001), and its quality was assessed using the AIC (AIC = 57669.6). Finally, for the 200 m races, gender (x^2^ (1) = 1099.5, p < 0.001), age group (x^2 ^(11) = 7765.4, p < 0.001), and the interaction between gender and age group ((x^2^ (11) = 32.7, p = 0.001) all had significant effects. The model was again superior to the null model according to the Omnibus test (p < 0.001), and its quality was assessed using the AIC (AIC = 47259.2). The differences between age groups of the same gender and between genders for each swimming distance are presented in Figure 3(Fig. 3).

## Discussion

This study aimed to explore the trends in participation and performance among master's butterfly swimmers in 50 m, 100 m, and 200 m races held in FINA competitions between 1986 and 2019, considering nationality, gender and age groups. We hypothesized that the USA would produce the fastest swimmers, men would have better times than women in the same age group, and younger categories would have better times overall. 

The main findings of the present study were (i) the country with the highest number of women swimmers in the top 10 fastest times by year in all butterfly distances was Germany, (ii) the country with the highest number of men swimmers among the top 10 fastest times per year in all butterfly distances was the USA, (iii) Germany, the USA and Great Britain were the only countries that had swimmers in the top 10 for all women butterfly distances and (iv) Germany, the USA and Great Britain were the only countries that had swimmers in the top 10 for all men butterfly distances, (v) men were faster than women for all distances and (vi) swimmers in younger age groups were faster than swimmers in older age groups.

Several countries, including the USA, Germany, Great Britain, Russia, Brazil, and Italy, produced top-performing butterfly swimmers. Among these countries, the USA, Germany (mostly as East Germany in the past), Great Britain and Russia (also considering the performance of the Soviet Union in the past) were in the top 10 list of the overall medal table of Summer Olympics (https://olympics.com/en/olympic-games; accessed on 16 May 2023). The outstanding infrastructure for swimmers could be one of the reasons for the success of these nations (Linthorne, 2007[47]). Moreover, these countries may have a shared interest in promoting master swimming (Linthorne, 2007[47]).

In the United States, U.S. Masters Swimming (USMS), provides a wide range of resources for swimmers, including a calendar of events, assistance in finding clubs, articles and videos to enhance performance, and a vast library of workouts for various training styles (https://www.usms.org/). Similarly, in Germany, the German Swimming Federation (Deutscher Schwimm-Verband or DSV) offers a comprehensive list of swimming clubs and races for age group swimmers (https://www.dsv.de/home/; https://www.dsv.de/masterssport/). Swim England Masters in Great Britain provides access to various races, links to the masters' community, technique tips, and nutrition advice (https://www.swimming.org/masters/). In Russia, the All-Russian Swimming Federation (Всероссийская федерация плавания/ ВФП) focuses on elite swimmers while providing support and promotion for youth athletes (https://russwimming.ru). The Brazilian Confederation of Aquatic Sports (Confederação Brasileira de Desportos Aquáticos/ CBDA) prioritizes pool swimming, open-water swimming, artistic swimming, water polo, and diving, while master swimming is a lower priority (https://www.cbda.org.br/). Finally, the Italian Swimming Federation (Federazione Italiana Nuoto/FIN) has a dedicated section for master swimmers (FIN, federnuoto.it/home/master). Therefore, it is evident that these countries are actively promoting master swimming and swimming infrastructure. These policies play an important role in enabling swimmers high availability of swimming pools which contributes directly to the number of workouts and swimmers' performance in these countries. 

It was possible to find variation of performance between men's and women's performance. This was expected due to biological determinants and development, where gender differences will affect peak performance. Women have a higher amount of adipose tissue than men (Siders et al., 1993[71]; Zuniga et al., 2011[83]) and proportionally more fatty tissue located caudally (McLean and Hinrichs, 1998[52]). This causes differences in the center of buoyancy between men and women (McLean and Hinrichs, 1998[52]). Men have an advantage in possessing higher peak leg power than women (Doré et al., 2005[21]; Hübner-Woźniak et al., 2004[36]; Martin et al., 2004[49]) primarily due to their greater lean leg volume (Perez-Gomez et al., 2008[65]), and peak leg power and lean leg volume with increasing age (Doré et al., 2005[21]; Martin et al., 2004[49]). This advantage helps offset fatty tissue's buoyancy effects, giving men an advantage. One key difference between men and women in this respect is that men typically have a greater stroke length than women (Seifert et al., 2008[70]). Furthermore, high-level women would show larger distance per stroke but would be dependent more than men on increased stroke rates (Craig et al., 1985[18]). With a more stable and longer stroke length, a swimmer can achieve faster swimming (Morais et al., 2022[55]). The cardiorespiratory variation between genders also contribute to the performance differences. Men have larger lungs and airways which are wider than women, therefore men present higher lung volumes and capacities and lower airways resistance (Blair, 2007[10]). There are also multiple differences in the cardiovascular system, such as the greater left ventricular mass and chamber size that the men presented compared to women. Consequently, men's cardiac output is greater than women's, which generates a higher maximal oxygen consumption (V̇O_2_max), positively affecting sports aerobic performance (Bassett and Howley, 2000[8]; Huxley, 2007[38]; Legato, 2000[45]). The causes influencing the decline of performance in the aging process were extensively investigated (Hayflick, 2007[33]; Kitani, 2007[43]; Njajou et al., 2010[61]). Age-related performance changes have been documented in several athletic activities, and the underlying physiological mechanisms, including V̇O_2_max, maximal heart rate and blood lactate levels, have also been documented (Allen and Hopkins, 2015[2]; Wolfrum et al., 2013[78]; Zingg et al., 2014[82]). These changes can be attributed to various biological alterations that occur with aging, including changes in the structure and function of organs like skeletal muscles, the heart, vessels, and the brain (Faulkner et al., 2007[26]). Moreover, the maximal cardiac output decreases with advancing age (Hunt et al., 1998[37]; Julius et al., 1967[42]; Saltin, 1986[68]). Due to reductions in both maximal stroke volume (Ogawa et al., 1992[63]) and maximal heart rate, older endurance-trained athletes experience a reduction in maximal cardiac output compared to their younger counterparts (Ogawa et al., 1992[63]; Rivera et al., 1989[66]). 

As individuals progress from early adulthood, their maximal heart rate decreases at a rate of approximately 0.7 beats per minute per year, regardless of whether they are healthy and sedentary, recreationally active, or endurance exercise-trained adults (Tanaka and Seals, 1997[74]). The decrease in maximal heart rate is believed to be due to several mechanisms, such as slower conduction velocity, reduced responsiveness of the sinoatrial node to β-adrenergic stimulation (Fleg et al., 1994[30]), and decreased intrinsic heart rate (Jose and Collison, 1970[41]), among others.

The large elastic arteries become stiffer with age, increasing aortic input impedance and vascular afterload. This impedes the ejection of blood from the left ventricle during systole and reduces stroke volume during exercise (Chen et al., 1999[14]). This could contribute to the reduction in maximal stroke volume observed in older endurance-trained adults, as it increases the left ventricular afterload and aortic input impedance (Mazzaro et al., 2005[50]). 

It is well-known that V̇O_2_max decreases with age (Valenzuela et al., 2020[77]). The gradual loss of muscle mass and strength that occurs with aging is called sarcopenia, and it leads to a decline in functional capacity (Snijders et al., 2009[72]). V̇O_2_max reduction is the primary mechanism associated with declining performance among the three primary determinants of endurance exercise performance (Tanaka and Seals, 2008[75]). The reduction in the lactate threshold, the exercise intensity at which blood lactate concentration increases significantly above baseline, also contributes to the reduction in endurance performance with age, although it may be secondary to the decreases in V̇O_2_max (Tanaka and Seals, 2008[75]). Older swimmers exhibited lower V̇O_2_max, higher blood lactate concentration, and higher alactic and lactic energy expenditures, while the relative alactic contribution decreased, and the aerobic contribution increased (Hellard et al., 2018[34]). This may also explain why the swimming time of older athletes is increasing, especially in a sport that, in addition to being very technical, is extremely demanding from an energy point of view. Determining the age of peak swimming speed could provide an estimate of when elite swimmers start experiencing a decrease in their swimming speed.

One of the most striking effects of age is the involuntary loss of muscle mass, strength, and function, termed sarcopenia (Lexell, 1995[46]). Because muscle mass decreases approximately 3-8 % per decade after the age of 30 and this rate of decline progresses even further after the age of 60 (Deschenes, 2004[20]; Holloszy, 2000[35]), a progressive regression of performance in older age groups is noticeable.

This study allowed us to highlight some important information about the origin of the fastest master butterfly swimmers (Germany for women and the USA for men). However, this research has several limitations related to the design: (i) this is only a time-based research analysis; (ii) no biomechanical or physiological determinants were analyzed; (iii) no information about the training conditions was considered; (iv) it is possible that the same athlete who performed among the top 10 over multiple years had his/her performance included multiple times in the analysis, which could affect the independence of the observation but cannot be excluded due to the format of the database; (v) the age group analysis is a retrospective cross-sectional observation, therefore decline in performance in different age groups should be interpreted with caution as they involve distinct athletes.

Future studies should compare training variables (such as volume, intensity, and frequency), competition history and coaches' expertise. Finally, biomechanical and physiological variables comparisons will allow one to better understand why some regions develop top-performing swimmers.

## Conclusion

Germany had the fastest master butterfly swimmers among women across all distances, while the USA had the fastest master butterfly swimmers among men for all distances. All distances were covered faster by men compared to women, and younger swimmers were quicker than older swimmers. This study's findings can help identify the specific countries of origin of the top-performing master butterfly swimmers and lend themselves as a basis for future studies to identify the contributing factors to this success, which can facilitate the development of policies, more effective training programs and coaching strategies. Overall, swimming remains a popular sport with a rich history and a bright future. As technology and science continue to advance, we will likely see further improvements in athletic performance and new records being set.

## Declaration

### Acknowledgments 

Not applicable. 

### Author Contributions 

KW drafted the manuscript, AlS performed the statistical analysis and prepared methods and results, WA and SM obtained the data, and MSA, SM, PF, IC, PN, ArS and BK helped in drafting the final version. All authors read and approved the final manuscript.

### Funding 

No funding was used to support this research or the preparation of the manuscript.

### Availability of data and materials 

The race data were obtained from the official website of World Aquatics, former FINA (Fédération Internationale de Natation) (www.worldaquatics.com). We obtained full data from all World Masters Championships held between 1986 and 2019 (www.worldaquatics.com/masters/archives/masters-archives). From each swimmer, the year of competition, first name, last name, age, age group, stroke, and distance were recorded. Due to the enormous data, we restricted to butterfly.

### Ethical approval 

This study was approved by the Institutional Review Board of Kanton St. Gallen, Switzerland, with a waiver of the requirement for informed consent of the participants as the study involved the analysis of publicly available data (EKSG 01/06/2010). The study was conducted in accordance with recognized ethical standards according to the Declaration of Helsinki adopted in 1964 and revised in 2013. 

### Consent for publication 

Not applicable. 

### Competing interests 

The authors declare no competing interests.

## Figures and Tables

**Table 1:**
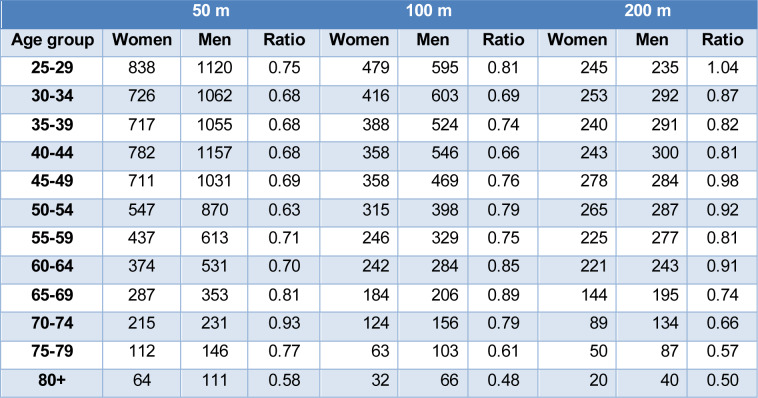
Women to men ratio in each age group and distance

**Table 2:**
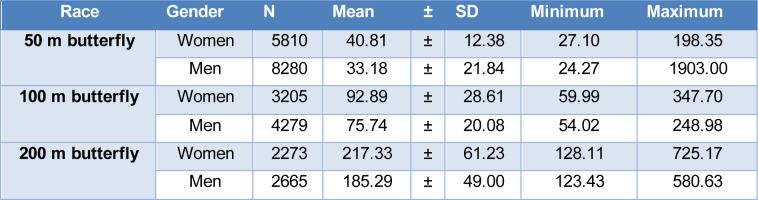
Mean time in all butterfly distances

**Table 3:**
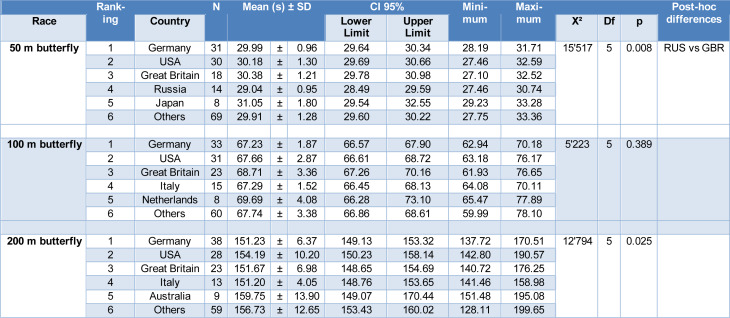
Significant differences between nationalities with women athletes in the top 10 in all butterfly distances

**Table 4:**
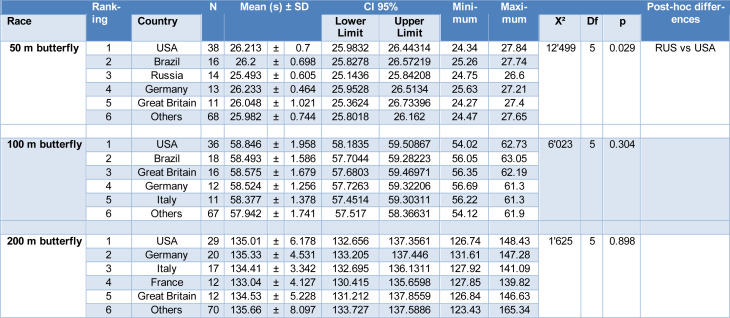
Significant differences between nationalities with men athletes in the top 10 in all butterfly distances
